# Playing-Side Analytics in Team Sports: Multiple Directions, Opportunities, and Challenges

**DOI:** 10.3389/fspor.2021.671601

**Published:** 2021-07-05

**Authors:** George Foster, Norm O'Reilly, Zachary Naidu

**Affiliations:** ^1^Graduate School of Business, Stanford University, Stanford, CA, United States; ^2^International Institute for Sport Business & Leadership, Lang School of Business & Economics, University of Guelph, Guelph, ON, Canada; ^3^Student, Stanford University, Stanford, CA, United States

**Keywords:** analytics, sabermetrics, player, professional sport, MLB, NBA, NFL, NHL

## Abstract

This paper describes developments in the player-side analytics in major team sports. We take a decision-making lens to the role of analytics in player decisions by general managers and coaches. We outline key accelerators and inhibitors to the wider adoption and acceptance of data analytics playing a greater role in the decisions of clubs.

## Introduction

The rise of data analytics is impacting many areas of business and society. Education institutions are increasingly recognizing this shift and are seeking to help meet the continued growth in demand for talent in this area^1^. In the sports landscape, both the playing-side (“on-the-field”) and the business side (“off-the-field”), reflect an increasing adoption of data analytics. This paper focuses on data analytic investments in the playing side of sporting clubs and the broadening areas of its applications. The debate in this area has shifted from whether analytics can be fruitfully implemented to the speed and depth of the appropriate investments to make. Although not explored in this paper, we note that a similar transformation is occurring in many areas on the business side such as fan engagement, ticket pricing, and sponsorship evaluation.

Sports for many decades has always had a strong quantitative aspect. Games are won or lost on the basis of numerical scores such as goals scored and runs scored. Rankings of individual players likewise has long been based on quantitative metrics such as goals scored, assists, passes caught and strikeouts made. Data analytics takes such quantitative information, and increasingly also qualitative information, as inputs to then rigorously promote decisions being made on a more informed basis with a higher likelihood of desired outcomes. Increasingly data analytics is also being used to probe hypotheses about which actions are likely to lead to more desired actions as well as to examine the validity of existing “conventional wisdoms” or traditional approaches to making playing side decisions. As noted in several parts of this paper, a higher probability of desired outcomes does not rise to certainty. A good decision based on a higher probability of desired outcomes can still have an outcome that is a less preferred one.

Section Moneyball Highlights the Potential Value of Data Analytics of this paper briefly highlights the origins of Sabermetrics and the derivative role of *Michael Lewis's 2003 Moneyball:*^1^ *The Art of Winning an Unfair Game*^2^ in building greater recognition of the potential value of using rigorous data analytics in player-side decision making in team sports. It also highlights why the batting side of baseball was an early attractive platform to showcase the potential value-add of data analytics to these decisions. Sections Player Squad Assembly Strategies and Data Analytics to Health and Fitness and Data Analytics discuss areas where analytic advances and adoptions are increasingly part of playing-side decision making: (i) player squad assembly strategies, (ii) pre-game and within-game strategy, and (iii) health and fitness. Section Growth Accelerators and Growth Inhibitors of Broader and Deeper Analytics Adoption in Player-Side Decision Making examines growth accelerators and growth inhibitors of analytics implementation in sports. Section MLB Case Study Highlighting the Sustained Success of the Oakland Athletics presents a quadrant analysis of MLB clubs based on club total player payroll and club regular season wins over the 1998–2019. This analysis highlights the success of clubs with relatively low payrolls and relatively high wins, notably the Oakland Athletics and later also the Tampa Bay Rays. This on-the-field success has been an important growth accelerator/stimuli to placing player-side analytics higher on the agenda of many professional clubs (and colleges) and their teams. Section Evidence on Diverse Composition of Personnel in Data Analytics Groups of MLB Clubs examines the structure and composition of MLB club analytics groups and Section Summary provides a brief summary.

Many of the specific advances in playing side analytics are rarely are placed in the public domain by individual clubs. While some generic advances are in the public domain, many innovative applications are occurring behind the curtains of clubs with the aim of maintaining any competitive advantage for those making those advances.

## Moneyball Highlights the Potential Value of Data Analytics

The conventional wisdom in sports economics is a positive (but far from perfect) correlation between player payroll and on-the-field winning. For example, annual editions of *Deloitte Annual Review of Football Finance* showcase a strongly positive correlation occurring in the English Premier League (EPL) between club investment in players and on-the-field performance. What attracted much early attention to analytics was evidence that disciplined application of analytics for player evaluation and team squad building could increase the likelihood that a low payroll club could achieve a sustained above expected number of wins on the playing field. Such an outcome would be counter to conventional sports economics predictions. An important heritage to this application of analytics came from early developers and advocates of Sabermetrics in baseball.

Earnshaw Cook (author of *Percentage Baseball*, MIT Press, 1964) and Bill James (whose Baseball Abstracts series started in 1977) are two recognized early pioneers of sport analytics. The term Sabermetrics, reportedly coined by James, comes from work at the ***S****ociety for* ***A****merican* ***B****aseball Research*, which was founded in 1971 to promote analysis of baseball through statistical means. These early advocates emphasized evaluating players on their ability to contribute to winning as opposed to a traditional metric such as batting average. For many years, this early work on Sabermetrics appeared to have limited impact within many professional baseball clubs. The Oakland Athletics were an early explorer of data analytics when a 1995 change in ownership led then General Manager Sandy Alderson to explore some sabermetric based approaches aiming to build a competitive playing squad with a relatively low payroll. Billy Beane, a playing side executive at the Oakland Athletics, was later to embrace this sabermetric approach.

Beane was the subject of Michael Lewis's 2003 *Moneyball*: *The Art of Winning an Unfair Game* which quickly became a best seller. Beane joined the Athletics in 1990 as a Scout and later became Assistant General Manager under Sandy Alderson. He became General Manager in late 1997. *Moneyball* highlighted how Beane used metrics such as on-base percentage^3^ and slugging percentage^4^, as opposed to traditional metrics (such as batting average and stolen bases), to identify players that were undervalued relative to the contribution they could make to the Athletics winning games. Over time, Beane continued to innovate, albeit not having so much of his analysis appearing in the public domain^5^. By 2011, when the Moneyball film was released, the strategy of seeking to identify undervalued assets in sports was gaining more widespread recognition. A key underpinning to this strategy is that player evaluation needs to assess (i) player on-field ability to contribute to winning, (ii) player ability to reliably be available to play the full contingent of games in the regular season and all playoff games, and (iii) the salary cost of attracting and retaining that player to the club.

### Baseball as an Attractive Early Use Case of Data Analytics for the Playing Side

In the two decades since the publication of the *Moneyball* in 2003, there has been widespread adoption of analytics across many areas of the playing side-functions of sporting clubs as well as across many sports. The following four factors help explain differences across sports with respect to the adoption of analytics for talent evaluation and why baseball was an attractive early candidate to illustrate the value-add of data analytics.

First, whether a sport has on-the-ball action as central vs. a sport where off-the-ball action can be as equally important as on-the-ball action is a key difference. Consider evaluating a hitter in baseball or a batter in cricket, both key positions in playing side offense in these sports. In each case, the hitter (batter) is the sole person facing the ball and actions by others on the team cannot assist the hitter (batter) ability to hit the ball. In contrast, consider a forward on a soccer or hockey (field or ice) team. Here, other players on the team can draw defenders into positions that enable that forward to have a higher likelihood of scoring. Similarly other players on the team can “assist” the forward to score by the timing and placement of their passes to that forward. Sports where both off-the-ball action and on-the-ball action are important require a sizably more complex tracking of interactions during the game to enable analysis. In these cases, the performance of the player is a joint product with other players on the field.

A second key difference lies in sports that have a stop-start rhythm vs. sports with a continual flow rhythm. Data collection is more well-defined in a stop-start sport than in a continual flow sport. There is a spectrum of sports on this dimension. At one extreme is baseball, cricket, and American football (gridiron). Here, there are defined stops that occur before another on-the-field interaction occurs at which a scoring (or another outcome) could occur. In baseball (or cricket) after each pitch is thrown (or ball is bowled), there is a stop before the next pitch is thrown (or ball is bowled). In gridiron, after each play the game is stopped until the offense team makes the next play. The other extreme is soccer and hockey where the ball (or puck) can progress forward or backwards in a continuous way without any stoppage in play. Here, tracking actions by any one player is a more complex challenge than with stop-start sports where there are well-defined natural data collection points at each stop point. While advances in tracking movements in a game is reducing this factor, it is one explanation for the slower adoption of rigorous analytics in continual flow sports.

The third factor is sports with many games per season vs. sports with few games per season. The more games per season, the more data points to analyze. MLB has 162 regular season games, NBA and NHL has 82 regular season games, English League Premier has 38 regular season games, and the NFL has 16 regular season games. The larger the number of data points the more reliable the findings of the analytic analysis and the less the impact of random or idiosyncratic events.

Fourth, sports with many scoring events per game vs. sports where there are few scoring events per game is an important distinction in data analytics. Soccer and ice hockey can result in games with just a few goals per game and in the extreme zero goals. Baseball games often average between 4 and 5 runs per game per team. Professional and college basketball games often have more than 50 scoring moments (successful two or three point shots). Given that scoring is the metric on which wins and losses are based, the higher the number of scores in a game the higher the number of key data points that an analytics approach can examine. While many more data points exist for within-game events in each of the above, the more defining points as regards the object of winning are the actual scoring events and the related leadups to those events.

The sports which facilitated early opportunities for data analytics as regards evaluating on-field contributions to winning are sports that have (i) on-the-ball action as central, (ii) are of a stop-start nature, (iii) have more than a few scoring points per game, and (iv) have a large number of regular season games. Baseball is highly attractive as regards (i), (ii), and (iv). Thus, it is no surprise that the focus of *Moneyball* and other much other early discussion on analytics in sport was on offensive hitting analytics in baseball.

Studies using data from MLB Media Guides highlight the growth of the data analytics function in MLB clubs. Lindberg and Arthur (2016) “took three snapshots of MLB ‘quant' staffs” by studying the 2009, 2012, and 2016 Media Guides of clubs and “cached online directories.” They defined a “quant” as “a baseball operations employee who spends a majority of his or her work hours either directing a quantitative department or doing statistical research, data processing or programming to support the team's analytical efforts.” They reported growth in positions as follows: “In 2009 a total of 44 employees fit our ‘quant' definition, and at least a third of teams had yet to assign a single full-time employee primarily to statistical work.” By 2012, the number had climbed to 75, and only four teams had no “quants.” Just 4 years later (in 2016), the analyst count has more than doubled again, to 156, and as of 2016, no team operates without some semblance of an R&D department” (Lindberg and Arthur, 2016).

In the next three sections we discuss areas where analytic advances and adoptions are increasingly being part of playing-side decision making across multiple team sports.

## Player Squad Assembly Strategies and Data Analytics

Decisions related to assembling a team playing squad aim to build a squad that will best achieve the objectives of the team owner/stewards. These objectives typically have a win aspect (i.e., “on-the-field” performance) given the constraints of a total player payroll budget. Decisions in this area focus on the expected future performance of players. General managers making these decisions are increasingly accessing data analytics, although with sizable variation across clubs in the importance given to analytics information. An key input here is information on the past and current performance of players being evaluated to be on the squad for the period under analysis.

Depending on the league and the sport, there are multiple categories of players being evaluated: (i) youth, (ii) draft-selections, (iii) existing players under contract, and (iv) free agents. As you progress from (i) or (ii) to (iii) and (iv), there is a richer and more reliable data base to evaluate past and current player ability. Moreover, there is higher uncertainty in (i) and (ii) when predicting how a player will perform in the future at the highest level of the professional club game. As with all analytics based on past data, there remains uncertainly about whatever past and current ability is identified for a given player in each of (i) to (iv) will progress or sustain going forward and for how long. Notably, many players' on-the-field performance declines later in their careers.

Soccer clubs in many leagues have youth academies. An extensive 2008 study of multiple soccer youth academies by the European Club Association reported that “the goal for most clubs regarding their youth academy is to develop players for professional soccer, in particular for their own first team.”^6^ Early data analysis often focused on existing elite players and looking backwards at their profiles including evidence of their proficiencies at various ages plus profile data about their parents. One early limitation here was that there was often minimal systematic data on youth predicted to become elite players but who were later exited out of the academies. Over time, the academies could develop a richer data base about youth in their academies and more effectively examine how contemporary information could predict subsequent success or lack thereof. Manchester United's Youth Academy is often cited as a success, given that multiple players on their first team were graduates^7^ Oft-cited examples include Bobby Charlton, Ryan Giggs, David Beckham, Paul Pogba and Marcus Rashford. For entry into a youth academy, the forecasting horizon is many years ahead and the objective (i.e., early identification and then development of first team members) being predicted is a very low probability outcome.

College recruitment of North American high school students, especially for basketball and football, is an area where colleges are increasingly investing in data analytics to identify and prioritize recruits to target. *Hudl* exemplifies the growth in analytics service companies that many teams have subscribed to^8^. It was founded in 2005 and provides information based products for multiple player side decision areas by professional clubs and colleges for many sports including football, basketball, and soccer. *Hudl* invests in developing data bases and in providing a ranking of prospects that colleges can use to complement scouting done by coaches observing live as well as recordings of games by high school players^9^. Data bases for youth often need to be interpretated very cautiously as there can be higher variation in the abilities of athletes on opposing teams than exists as players advance to being professionals and their on-the-field competition has more comparable levels of ability.

Companies such as *Hudl* are important growth accelerators for sporting clubs expanding their use of data analytics. By having multiple clients, it can make a larger investment in many areas of data analytics that are beyond the budgets of most sporting clubs. Acquisitions of competitors (such as *Krossover* in 2019) has further enabled *Hudl* to be an even larger force in the player side data analytics landscape.

### Building a Squad That Captures Where the League Is Evolving

One frequent metaphor used in business is the famous expression oft-quoted to NHL hockey great Wayne Gretzky (Kirby, 2014): “Skate to where the puck is going, not where it has been.” This metaphor as regards squad building captures the notion that when assembling a playing squad, it is important to understand shifts in the structure of the game. NBA basketball has been at the forefront in this area, fueled in part by all NBA arenas having many cameras above the court, a rich data base of multiple scoring attempts and points from many games, few players on the court to track, and innovative analysts. For many years, there was a high focus on building basketball squads around dominant centers who made many two point shots close to rim (so-called “in the paint” shots). It is only since the 1979–80 season that a three-point shot has been scored in the NBA long after it had been introduced in the smaller American Basketball League in 1961. The NBA was reported to “consider it gimmicky for years. The NCAA was even slower to adopt the rule” (Wood, 2011). More recently, three point shooters are being given higher priority in squad assembly (Garcia, 2020). The standout three-point records of Steve Nash with the Phoenix Suns in the 2000's and Stephen Curry and Klay Thompson with the Golden State Warriors in the 2010's are considered important stimuli to greater value being given to successful three point shooters in playing squad assembling decisions.

**Figure 1:**
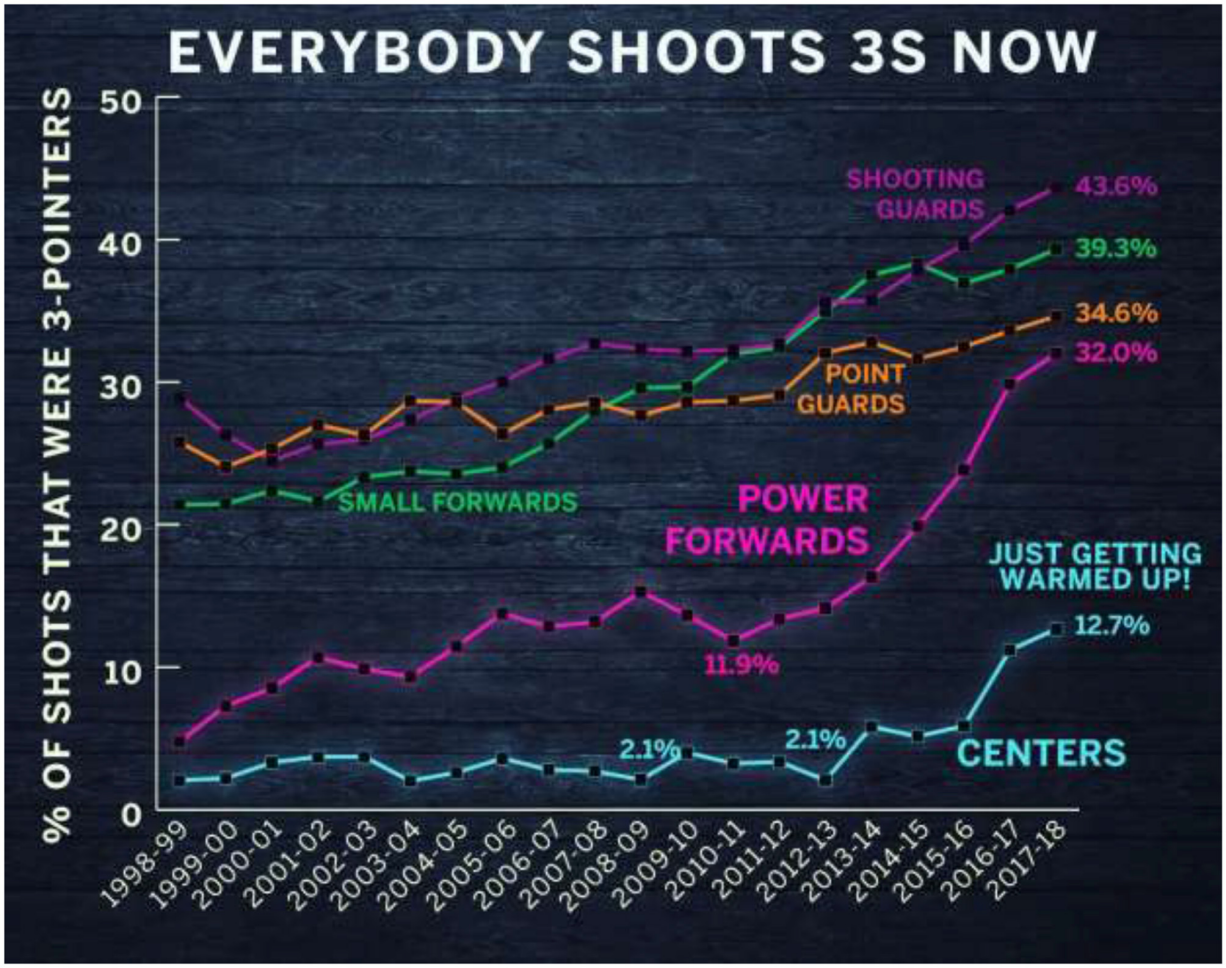
Analytics analysis of shifting structure of NBA games—an increasing shift to 3-point shots. Source: Reprinted with permission of Goldsberry (2019).

Kirk Goldsberry, a leading thought leader in basketball analytics, has showcased the changing dynamics in basketball in his recent book ***Sprawball***(Goldsberry, 2019). He documents the progressive increase in the percentage of NBA shots that were three-point shots since the 1998–99 season, reaching 35% in 2017–18. Figure 1 (below) depicts Goldsberry's presentation of the shift by different basketball positions in their relative frequency for taking three point shots.

Underlying what is presented in Figure 1 is an analysis of every shot taken by every NBA player each year with tags as to their court position and whether they were within the 2-point Arc vs. in the three-point zone (i.e., beyond the three-point line). The changing compensation of the top 10 highest paid NBA players from 2011–2012 to 2020–2021 further reflects and supports the shift to players that have a track record of high three-point success and the move to less representation by Power Forwards and Centers (i.e., players normally focused on two-point scoring). Based on an analysis using Spotrac public data, the percentage of top ten compensated NBA players across the five positions is found as Table 1.

**Table 1:** The percentage of top 10 compensated nba players across the five positionsm.

	**Point**	**Shooting**	**Small**	**Power**	**Center**
	**guard**	**guard**	**forward**	**forward**	
2011–2012 to 2015–2016	16%	14%	24%	30%	16%
2016–2017 to 2020–2021	38%	18%	28%	10%	6%

*Data Source: Spotrac (accessed June 09, 2021)*.

Pre-game and within-game analysis now often uses extensive analytics-based reports on the relative ability of individual opposing team players to successfully take three-point shots, and how that differentially varies across those players by the location outside the three–point line where each player takes their three-point shot attempts.

### Predicting the Future Performance of Athletes: Pattern Recognition Based on Data Analytics

Predicting the future performance of an athlete can have varying levels of uncertainty. Decision makers gain insight by building evidence based pattern recognition skills. Data analytics are an important pillar to building more reliable pattern recognition skills and can be an important counter to excessive reliance on anecdotes or the personal biases of playing-side decision makers. A key role is to increase the likelihood that players with superb ability to contribute to winning are incentivized and appropriately rewarded (within which extant league rules allow) and those with much less ability are not overpaid and are not given long term contracts that over time can become increasingly burdensome to the club.

An oft used expression when evaluating players for a squad is “the most important ability is availability.” Underlying this comment is the notion that, with all other things being equal, the higher the percentage of games a player is available to perform at peak performance levels, the more valuable that player is to a squad. The reasons why a player may not consistently be available to perform at this highest level include: (i) expected impact of aging, (ii) physical injuries, (iii) failure to maintain high levels of health and fitness, (iv) attitude and mental state of mind issues, (v) sidelined due to legal or league penalties, and (vi) sitting out due to contractual disputes.

### Aging Curve Data Analytics Evidence

**Figure 2:**
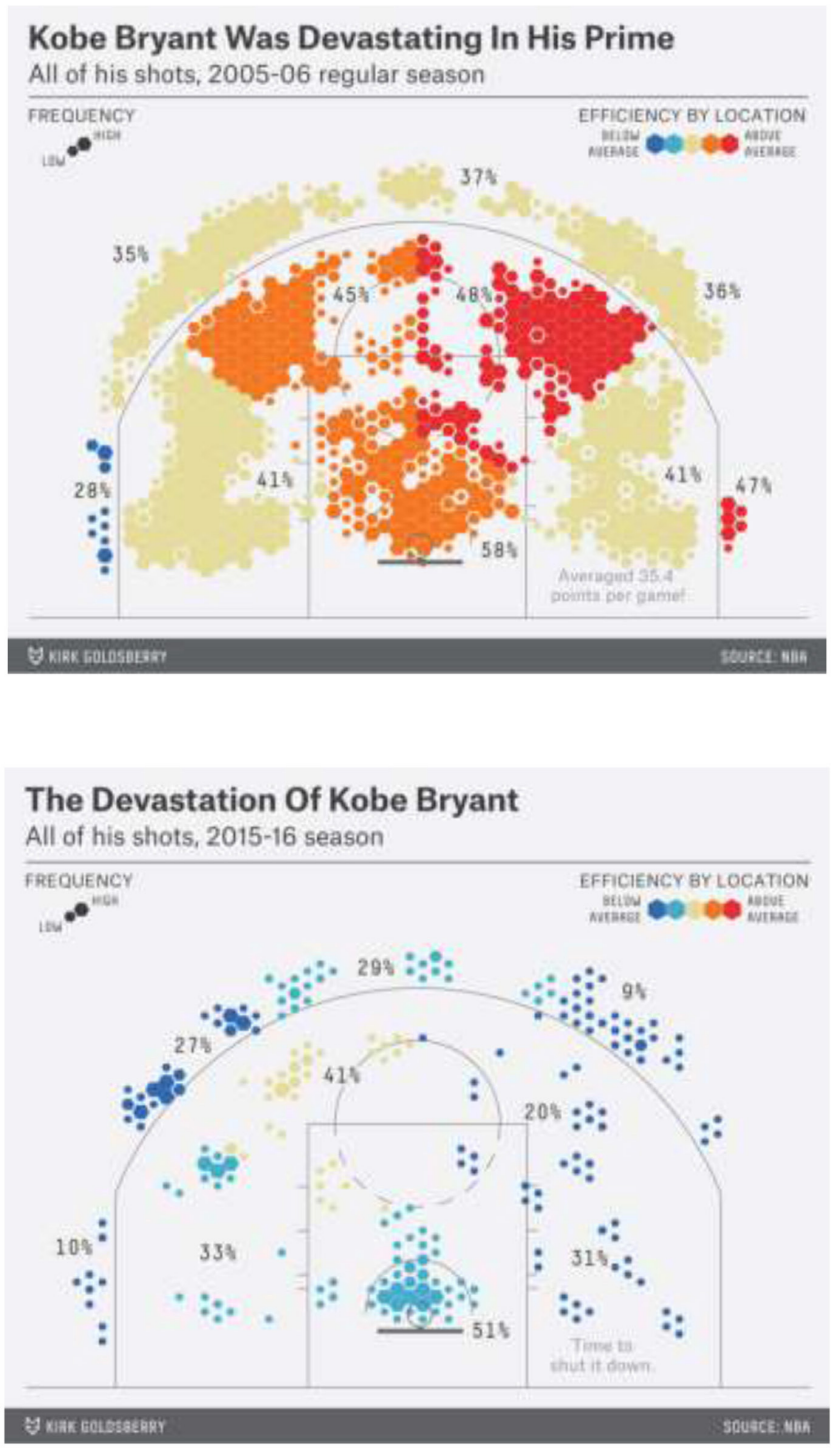
Tracking athlete performance decline: shooting efficiency analysis of Kobe Bryant 2005–2006 vs. 2015–2016. Source: reprinted with permission of Goldsberry (2019).

General managers typically will be negotiating multi-year contracts with players, especially with elite players whose contracts have sizable financial amounts at stake. An important role of analytics is gaining insight into the expected future playing performance of a player based on a systematic analysis of many athletes and the riskiness of signing a given player to a long-term contract. The shooting efficiency charts in Figure 2 below highlight from Goldsberry's (2019) analysis of the contrasting skill levels of Kobe Bryant in 2005–2006 (at age 27) and in 2015–2016 (at age 37). The specific percentage points are reported in Table 2.

**Table 2:** Kobe bryant shooting accuracy: 2005–2006 vs. 2015–2016.

**In-the-paint**		**Shots within arc (2-Points)**				**Shots beyond arc (3-Points)**				
	**#1**	**#2**	**#3**	**#4**	**#5**	**#6**	**#7**	**#8**	**#9**	**#10**
2005–2006	58%	41%	45%	48%	41%	28	35	37	36	47
2015–2016	51%	33%	41%	20%	31%	10	27	29	9	M

*Goldsberry (2019)*.

Bryant's shooting efficiency declines in every shooting location on the court (left to right on the shooting efficiency chart, Table 2). Note that M means minimal shots taken. The locations are #1 in the Paint, #2 to #5 Within the Arc: 2-Point Shots (#2 Short Corner, #3 Wing, #4 Elbow, and #5 Low Post or Block) and #6 to #10 Beyond the Arc: 3-Point Shots (#6 Corner, #7 Wing, #8 Point, #9 Wing, and #10 Corner). The Arc refers to the 3 point line. M means minimum data (shots) available.

Basketball-Reference.com reports that in the three sessions from 2003–2004 to 2005–2006 Bryant averaged 29 points per game and played an average of 70 out of the 82 regular season games. However, in the three seasons a decade later, from 2013–2014 to 2015–2016, he averaged 18 points per game and played an average of 36 regular season games. Bryant's lower productivity per game and lower availability per season as he aged illustrate the gravity effects of aging that is well-documented across most athletes. Athletes who defy this gravity effect of aging well-beyond expected norms (such as Tom Brady in gridiron and Lebron James in basketball), while rightfully celebrated, are outliers and exceptions to what age-based analytics documents.

Consider the San Francisco Giants decision in December 2006 to sign the then 29 year old pitcher Barry Zito to a 7 years, $126 million guaranteed contract. After Barry Bonds stopped playing with the Giants at season end 2007, Zito became the highest paid Giants player from 2008 to 2010. In his seven years at the Oakland Athletics prior to signing with the Giants, Zito (aged 22–28 years at the time) won 102 and lost 63 regular season games, for an impressive win percentage of 61.8%. Analytics can provide input into the risks of a Zito type contract. For example, one published study (Ng, 2017) modeled MLB player performance as a function of age, experience and talent. The data analyzed included 5,754 seasons for 562 batters and 4,767 seasons for 489 pitchers and. Ng's (2017) findings were: “Peak physical age for hitters and pitchers are 26.6 years and 24.5 years, respectively, when holding experience constant. With increased experience, batters peak near age 29, while pitchers peak near age 28.” Unfortunately for the San Francisco Giants, Zito's performance followed (if not below) that predicted from systematic data analysis of many other pitchers over many seasons. In his seven years at the Giants, Zito (age 29–35 years) won 63 and lost 80 games for a win percentage of 44.1%. He had averaged 222.75 innings pitched per year from 2001 to 2006 at Oakland whilst at the Giants he averaged only 162.53 innings pitched per year. A comparable example from the MLB batting side is the 2011 decision by the Los Angeles Angels to sign the 31 years old Albert Pujols to a 10 year contract guaranteed for $240 million. In his prior 11 seasons with the St Louis Cardinals (age 20–31 years), Pujols averaged 40 home runs, 120 runs batted in, and played in 155 out of the maximum 162 games per season. From 2012 to 2019 with the Angels, he averaged 26 home runs, 99 runs batted in and played in only 140 games per season. In May 2021, the Angels waived Pujols before his contract ended. He had batted 0.198 in the first 24 games of the 2021 season.

One red flag for signing a long term contract with a pitcher older than 28 or a batter over 30 is the data analytics evidence (Ng, 2017) of how the performance of athletes typically declines as such athletes age. The second red flag is the systematic evidence of increased non-availability to play (even at a less than peak level) as players age beyond the average peak years for their position (Ng, 2017). For example, another study (Lindholm, 2014) reported a progressive decline in the number of MLB pitchers who can start 20+ games a season as they age beyond 28 years old. These so-called “aging curves” for pitchers (batters) are based on samples of many pitchers (batters) over many seasons. Whilst they may not be predictive of a specific pitcher (or batter), the sizable analytic evidence places the onus on the person deciding to sign the aging (beyond the average peak performance year) athlete to either (i) have credible evidence that the general aging curves are less applicable to the aging athlete being considered, or (ii) reduce the downside by writing a contract that is less exposed if the general pattern does subsequently apply to the athlete (such as a shorter contract or a contract with a lower guaranteed amount with bonuses for performance above expected).

### Contract Year Analytics Evidence

Many professional athletes will have multiple contracts during their playing careers. Near the end of an existing contract, General Managers (or Heads of Player Development) face the challenge of predicting the future performance of the athlete in a possible next contract. There is evidence from rigorous data informed studies across multiple sports that an athlete's performance in the final year of the first contract exceeds that which will occur in the first year of the second contract. The same phenomenon has also been documented for some sports between the final year of the second contract and the first year of the third contract (albeit the sample size for athletes with a third contract is sizably lower). This has come to be known as the “contract year phenomenon” (White and Sheldon, 2013).

An illustrative study by Hurley (2019) examined the scoring ability (defined by goals and assists) of forwards who made their debut in the National Hockey league between 2008 and 2019. This study examined scoring performance in the years surrounding their contracting from their rookie contract (often a 3 years contract) for those players who were offered a first non-rookie contract. Two measures of performance reported by Hurley (2019) were:

*Points Per game (PPG): (Total goals plus assists) divided by games played.
*Points Per TOI (PPTOI): (Total goals plus assists) divided by Time on the ice (TOI) in minutes.

**Figure 3:**
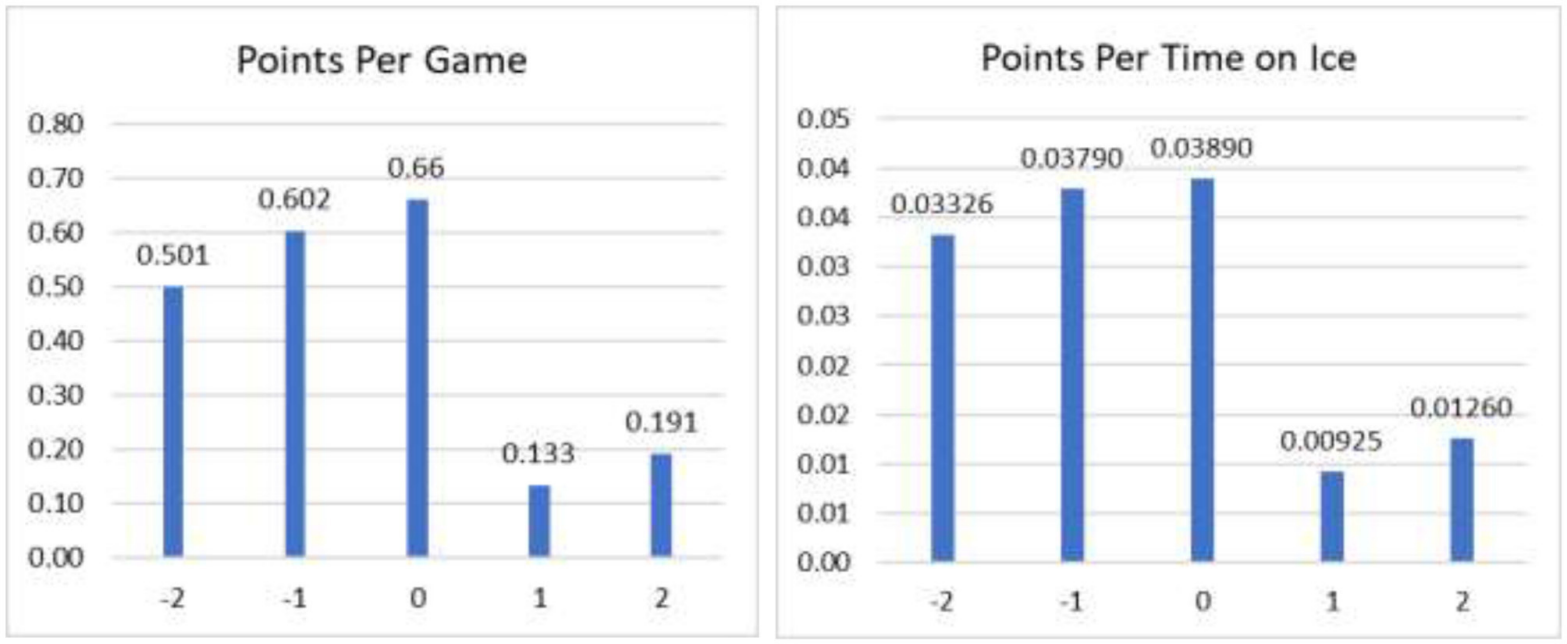
NHL player performance in rookie contract years and first-non-rookie years: 2008–2019. Source: Hurley (2019).

Figure 3 plots these two metrics for those players with performance data for a five-year period that encompasses the last 3 years of the rookie contract (called Year−2, Year−1, and Year 0) where Year 0 is the last year of the rookie contract and the first 2 years of the first non-rookie contract (called Years +1, and Year +2).

The dramatic pattern observed in Figure 3 is that the rookie in the last year of the first contract has very strong performance in scoring, but then performance in the second contract subsequently drops.

This contract year phenomenon has also been documented in baseball and basketball (White and Sheldon, 2013; O'Neill, 2014). One explanation is that players in the final year of their contract “pull out all the stops” in the year before their next possible contract and then revert more to their “normal” playing style (White and Sheldon, 2013; O'Neill, 2014). Such analytics-based information can assist a club general manager in several ways. One is in the period to write a subsequent contract. The default period for the first non-rookie contracts would be shorter (say 2 years) rather than longer and include incentive-based compensation. Another use is to include in contracts incentive-based clauses so that the player, as well as the club, shares the risks of subsequent drop-offs in the player's performance.

## Pre-Game and Within-Game Strategy and Data Analytics

Sporting clubs have long made quantitative assessments of the various levels of ability of their players and those on opposing teams. The advent of extensive data tracking for vast numbers of games has greatly facilitated more rigorous evidenced-based assessment of the abilities of individual players. It has also facilitated highlighting areas of player strengths that need reinforcing or weaknesses that either can be addressed or adjustments made in game strategy.

In many sports, key decisions are being made on a continual basis as a game progresses. Pre-game and within-game analytics is an area where much work has been conducted. Three examples here will be used to illustrate this application. These are (i) Fourth Down Decision-Making in College Football or the NFL, (ii) Field-Shifting Defensive Analytics in Baseball, and (iii) Basketball Defensive Strategy Analysis of Opposing Team. In each case, the role of pre-game or within-game analytics is to analyze data sets from many prior games to identify decisions that have a higher likelihood than other decisions of achieving preferred outcomes. The appropriate benchmark is whether decisions are made that increase the likelihood of preferred outcomes vis-à-vis decisions made without such data analytics.

### Fourth Down Decision-Making in College Football or the National Football League

One stream of football analytics examines how teams in varying contexts may have a higher likelihood of more points if they attempt the 4th down play over taking the field goal attempt. Higher likelihood does not guarantee that a touchdown will be made and coaches can vary in their willingness to assume the risk that a touchdown will not be made. The New England Patriots of the NFL are often used as an exemplar of the payoffs from being more aggressive in going for a touchdown than taking the higher likelihood field goal attempt^10^.

Charts called “4th Down Decision Analysis” built on two variables (distance to 4th down and field position) have long been inputs into decision making. Recent analytics research has examined multiple factors that affect the likelihood of getting the first down or the touchdown. For example, one college study of all games played by the 65 teams in the Power 5 Conferences from 2015 to 2018 reported that “distance to go, pass or run, the line of scrimmage, and the week of the season are significant factors in predicting a successful 4th down” (Blinkoff et al., 2020).

It is important to examine the context in which data is collected before generalizing from specific studies. Consider generalizing to the NFL from fourth down decision making evidence based on college data. Such evidence may be less generalizable to the NFL for several reasons. One reason is potential differences in fourth down rules between college and the NFL. For example, if you miss a field goal in college the ball for the opponent goes back to the line of scrimmage while in the NFL if you miss a field goal the ball for the opponent gets spotted where the kicker missed the kick. A second reason is that the success rate of kickers and punters in the NFL is typically higher than that in college games which is an input into analytics decision making in this area.

### Field-Shifting Defensive Analytics in Baseball

For many years, managers in baseball have recognized that hitters on an opposing team differ in where they hit the ball most frequently and how that may vary with different pitchers. The growing sophistication of MLB game tracking has increased the quality of information about the projection of balls and the characteristics of the pitcher's delivery (such as ball speed and batting box position of hitter) for each hit. Analytic charts are being developed based on tracking of the hitting profiles of the opposing team when facing either the pitchers they will be lined up the next game or those of other pitchers viewed as comparable. Using these charts, managers can place fielders in positions more likely to catch fly balls or keep base hits to a minimum. They can also experiment with different numbers of outfielders, such as a four outfielder defense as opposed to the long tradition of three outfielders. A four outfielder defense may have a better probability of an out or a restricted number of bases hit for a batter who hits more long balls and makes fewer ground hits through the in-field. For instance, the Houston Astros in 2018 shifted their fielders 43.5% of the plate appearances by the opposing team^11^. The growing use of this data is argued to be a major factor in baseball becoming more defensive driven and leading to lower scoring in many games^12^ A similar data analytic approach is being used by many cricket teams to guide field placement when different batters are at the wicket.

### Basketball Defensive Strategy Analysis of Opposing Team

Shooting charts (such as those for Kobe Bryant shown in Figure 2) for each of the players on a forthcoming opponent's squad are now a standard tool in basketball pre-game defensive strategy. The shooting charts of different NBA players illustrate how players have vastly different abilities that are important to consider in developing defensive strategies. Table 3 used data from Goldsberry (2019) to showcase the different shooting success rates of three NBA players.

**Table 3:** Shooting charts of curry, james and love for the 2015–2016 NBA season.

	**In-the-paint**	**Shots within arc (2-Points)**				**Shots beyond arc (3-Points)**				
	**#1**	**#2**	**#3**	**#4**	**#5**	**#6**	**#7**	**#8**	**#9**	**#10**
Stephen Curry	64%	M	M	M	M	43%	43%	48%	48%	52%
Lebron James	68%	41%	37%	37%	40%	24%	27%	28%	38%	28%
Kevin Love	53%	M	M	M	M	45%	36%	30%	38%	35%

*Goldsberry (2019): Curry (p. 65), James (p. 164), and Love (p. 168)*.

Coaches and players on the opposing teams gain much by anticipating the playing patterns of different players they will be defending against. Shooting charts like that in Figure 2 can also be used by coaches and trainers with their own players to highlight areas of strength of their squad that need reinforcing or weaknesses that should be strengthened.

Changes in the playing focus of individual players can increase their value to a squad. Consider Kevin Love in the NBA. He has sizably changed his playing strategy to better align with the heightened awareness of the value of quality three-point shooting. As noted in Table 4, his percentage of total scoring from successful three-point shots over the first eight seasons of his NBA career increased sizably.

**Table 4:** Kevin Love proportion of three-point shotting, 2008–2009 to 2015-2016.

**2008–2009**	**2009–2010**	**2010–2011**	**2011–2012**	**2012–2013**	**2013–2014**	**2014–2015**	**2015–2016**
2.8%	16.4%	20.6%	26.6%	30.9%	35.6%	41.1%	44.9%

*Goldsberry (2019), p. 167*.

For his first NBA season, Love played as a center and then from 2009 to 2010 as a power forward. As Figure 1 illustrates, Love's shift in his own game playing is part of a general shift by all players in the NBA to increase their percentage of three-point shots. This shift has been accompanied by increased training efforts by coaches, trainers and players on this now pivotal part of basketball games.

## Health and Fitness and Data Analytics

Health and fitness analytics has been a growth area in all parts of society. Multiple startups and existing companies have developed numerous products and tracking devices that monitor the health and fitness of individuals. The sporting industry has been an important arena where these devices have been developed. Areas included heart monitoring, body muscle strength, sleep patterns, and injury prevention and rehabilitation. Some examples that illustrate the pervasive impact of analytics in several of these areas are presented in the following sub-sections.

### Training and On-Field Tracking of Athletes in Soccer

An illustrative study that highlights athlete tracking with wearables is the work of Ravindranathan et al. (2019). This study used information provided by the Zephyr Bioharness device for 21 players of a soccer team during both practices and games for the entire season. The variables tracked included time (seconds), heart rate (beats per minute), breath rate (breaths per minute), speed (mile per hour), peak acceleration (G force), and GPS–Latitude, Longitude Co-ordinates (degrees minutes seconds). Large amounts of data was regularly collected from each player. With this data, the researchers could analyze patterns across athletes in their performance. Elite soccer clubs are now regularly receiving downloads of such information after each practice. This data highlights how athletes vary in their reaction to training routines. It has also red-flagged the potential for the over-training of athletes, beyond which their performance levels decline. One side benefit that several coaches have observed is the early warning signals of underperformance by players in training that have benefits in their decisions about player selection for forthcoming games and time in those games before they are substituted. Another benefit is that it is harder for players with an inclination to “shirk” hard training routines to remain undetected.

### Injury Propensity Determinants for MLB Pitchers and Early Warning Signals

Sports medicine has been a central part of the sports industry for many decades. Innovative use of analytics is advancing the ability of clubs to tailor training programs and playing time in games to have athletes perform at their peak levels and reduce the likelihood of serious injuries. An example is Hurley's (2020) study of the connection between MLB pitcher velocity and Tommy John Surgery. Starting in 2007 the MLB installed PitchF/x in each ballpark. This is a pitch tracking system “that measures the velocity, movement, release point, spin, and pitch location for every pitch thrown, allowing statisticians to perform detailed analysis” (Hurley, 2020, p. 13). Tommy John Surgery “replaces a ligament in the medial elbow with a tendon from somewhere else in the patient's body. A torn ligament typically comes from repetitive use of the elbow, such as making the violent motions involved in throwing a baseball” (Hurley, 2020, p. 16). This study examined all pitchers who threw at least 100 innings in a season for the 2007 to 2019 seasons. The joint findings were that (i) pitchers who threw harder had a higher likelihood of injury needing Tommy John Surgery, and (ii) pitchers who threw harder had higher compensation (Hurley, 2020). One implication here is that reducing the innings pitched per game by high-velocity pitchers can result in their careers being extended. Future analytic research here offers the promise of identifying factors at the individual pitcher level that enable early warning signals of the differential likelihood of individual pitchers on a squad for a torn ligament injury.

### Sleep Patterns of Athletes and In-Game Performance

Sleep research is a rapidly growing research industry in general, as well as for the sports industry specifically. Specific journals such as *Sleep Medicine Reviews*^13^ publish many studies in this area. There is a growing body of evidence that the one-size-fits-all approach (such as recommended minimum sleep time) ignores important differences across athletes (and people in general) in the impact on their performance and injury likelihood of both the length and type of their sleep patterns. Sleep mobile apps are increasingly being viewed to gain insight into this area of research.

While sleep research is a major area of medical research for the general population, athletes can differ in systematic ways which makes it important to consider such differences when counseling athletes on sleep behavior. Athletes come from a more narrower area of the general population age profile, are typically more healthy and are more active in rigorous training routines. In some cases, athletes can have travel schedules that are different, such as sleeping in different beds for road games and spending more time on airplanes. Athletes in the NBA and NHL can face extra sleep challenges with so-called “back to back games” (games in different cities on successive days) that athletes in sports that play on a one game a week schedule (such as NFL football) rarely face. Walsh et al. (2020) provide an extensive review of research and “expert consensus recommendations” on “sleep and the athlete.”^14^ An example of athlete specific factors to consider is a higher likelihood of concussion for some positions and for some athletes a higher level of anxiety before major games or when they have had a period of extended under-performance.

## Growth Accelerators and Growth Inhibitors of Broader and Deeper Analytics Adoption in Player-Side Decision Making

**Figure 4:**
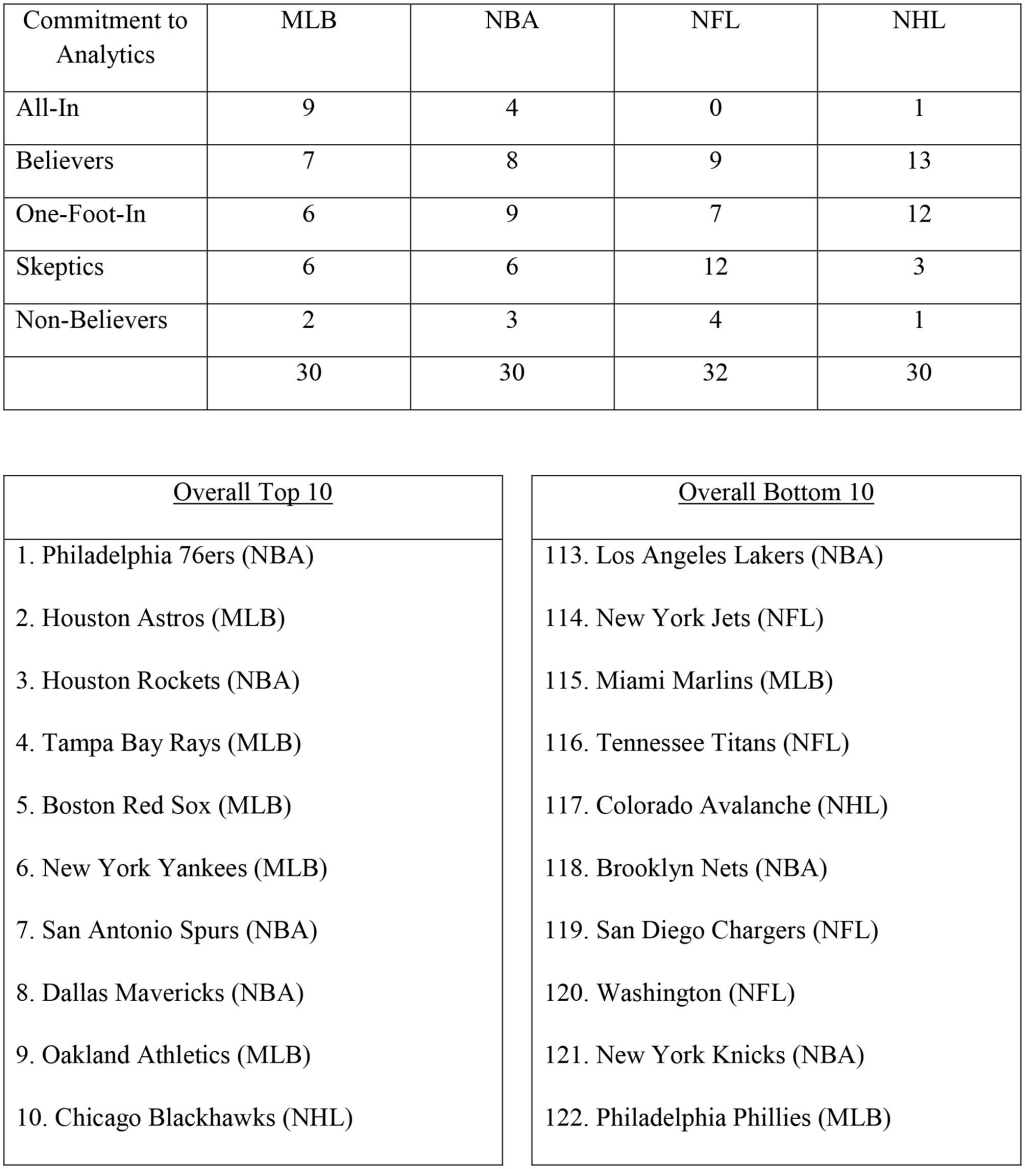
Analytics rankings of North American clubs in the MLB, NBA, NFL, and NHL by ESPN The Magazine in 2015.

The only known systematic ranking of the analytics groups of sporting clubs is the ESPN The Magazine's 2015 analysis of all 122 clubs in the MLB, NBA, NFL, and NHL. This article rated and then ranked the then 122 teams on the strength of each franchise's analytics staff, its buy-in from execs and coaches, its investment in biometric data, and how much its approach is predicated on analytics. Based on their analysis, clubs were also placed into one of five categories: “All-In,” “Believers,” “One-Foot-In,” “Skeptics,” or “Non-Believers.” A summary of this data is shown as Figure 4, which presents a summary of the clubs in each category by league, as well as the Top 10 and the Bottom 10. The ESPN study highlighted the vast differences across clubs in their perceived level of and commitment to analytics being an important part of player side decision making.

We now highlight some important growth accelerators and growth inhibitors for a broader and deeper adoption of analytics across sporting clubs.

### Growth Accelerators

This section describes growth accelerators for the broader and deeper adoption of analytics in player-side decision making. Multiple of these drivers apply not just to the sports arena but also to the expanding role of data analytics in many areas of commerce and society.

The first growth accelerator is *The Powerful Role of Leagues* which can involve multiple areas. One league role is mandating state of the art data analytics tracking of all games in a standard format that is quickly made available to all clubs for analysis. By covering the setup, costs and operation of such technology and data sharing, a league creates a more level playing field (and data access) across all clubs to key information for analysis. An example is the NFL's Next Gen Stats which is based on sensors being installed by the NFL in each stadium in the league. These sensors “capture real time location data, speed and acceleration for every player, for every play on every inch of the field. Sensors throughout the stadium track tags placed on players' shoulder pads, charting individual movements within inches.”^15^ The second league role is investment in new technologies and the evaluation of ongoing streams of new analytics products. A third role is league sponsored hackathons where third parties compete on best solutions to address league-wide problems in data analytics. Another role is a league having an internal consulting unit that highlights the best data analytics practices in the same way that some leagues have business side best-practices consulting groups (such as the NBA's Team Marketing & Business Operations (TMBO) unit) for their clubs.

A second accelerator is *Success Stories and Documented Innovations*. Success with a data analytic innovation can be an important prompt to its broader adoption in many areas. Section MLB Case Study Highlighting the Sustained Success of the Oakland Athletics of this paper documents the sustained on-field success of the Oakland Athletics. This success was an important prompt to other clubs adopting what they believed were some of the innovations underlying the Athletics on-the-field success. Injury and recuperation innovations that reduce the severity of player injuries or the time taken to recover have high value across all clubs and are likely to be quickly adopted by clubs and their players. Medical practitioners using these medical innovations have an economic incentive to publicize their specialties. Similarly, companies developing new tracking technologies that facilitate new ways of analyzing player movements on the playing field also have an economic incentive to broadly publicize their innovations.

Third, the *Mobility of Key Analytic Personnel* can accelerate data analytics adoption. Indeed, human capital mobility is a powerful way in which analytics adoption is fast-tracked by clubs wishing to expand their investment in analytics. Multiple examples of high profile analytics talent moving across MLB clubs illustrates this mobility. For example, Andrew Friedman went from the Tampa Bay Rays to Los Angeles Dodgers in 2014. A second example is Farhan Zaidi who moved in 2014 from the Oakland Athletics to Los Angeles Dodgers and then, in 2018, moved again to the San Francisco Giants. Multiple sports clubs are hiring analytics personnel from outside the sporting industry which has the extra upside of a “fresh set of ideas” being applied to many of the application area in sports.

*New Technologies and New Data Bases* is another accelerator. Major investments are being made by many companies and governments as well as the investment community (such as venture capitalists) in either new technologies (such as AI-Artificial Intelligence and Visual Recognition) or new applications of those technologies. The sports arena is just one of multiple areas where these investments are accelerating the adoption of data analytics. An example in the player-side of sports is the broader adoption of tracking wearables like the Zephyr Bioharness. These wearables enable clubs to build a large data base covering many player variables from every practice, training session or competition. Prior to the development and consistent use of such wearables by all players, the available date on player training was less structured, covered fewer variables, and was less reliable (in part due to inter-observer variability).

A fifth accelerator, *High Profile Conferences and Analytics Champions/Thought Leaders*, is a key engine in the broader growth of analytics. Knowledge transmission is one role of such conferences with their associated networking. Conferences in areas such as Artificial Intelligence and Biometrics showcase many speakers and companies. An important example in sports is the MIT Sloan Sports Analytics Conference^16^ which was started in 2006 and has built a tremendous industry reputation. A caveat here is that during these conferences and elsewhere, companies and clubs are often reluctant to extensively expose their analysis and findings in an attempt to retain a competitive advantage or to build revenues from products sold commercially.

*Academic and Corporate Research and Student Analytics Related Clubs* are a valuable accelerator of future adoption of analytics in general and sports in specific. Research on sports analytics is occurring at many universities as well as in sporting organizations and companies. Relatively new journals such as the *Journal of Sports Analytics* and *ASA: Statistics in Sports Section* illustrate the growing research activity in this space. Many universities now have sport analytics clubs that attract many students from diverse areas such as computer science, mathematics, statistics as well as economics, business, and sport management. Students in these clubs can be an attractive recruiting ground for clubs investing in sports analytics as well as a source of innovative ideas.

*Consumer led demand for analytic information* and insights is another growth accelerator where key external stakeholders find such information helps them make better decisions or have a richer viewing experience. For example, sports bettors are often avid consumers of data as they seek to improve their likelihood of winning. Sports broadcasters have been aggressive adopters of new technologies as they aim to increase viewer experience. For example, broadcasts of baseball games now showcase data on the speed of pitchers, the speed of runners, and the flight paths of balls hit. There can be a virtuous circle here where data analytic innovations that provide key insights for internal decision making by clubs are also built into broadcasts of sports. In some cases, the technical staff of broadcasters further extend the innovation in ways that clubs themselves then build into their own decision making.

### Growth Inhibitors

This section describes different growth inhibitors to the broader and deeper adoption of analytics in player-side decision making.

A major challenge facing sport analytics, is the *Cost of Building a Well-Resourced and Well-Functioning Analytics Groups*. Scarcity of talented seasoned personnel was a frequent blockage when some clubs made early commitments to scaling up their analytics function. While there has been an increase in the supply of such talent in recent years, such talent is highly attractive to a broader set of companies in other industries at compensation levels many sporting clubs are reluctant to meet. Billy Beane, in visits to Stanford University classes, frequently expresses concern that companies such as Apple, Facebook, and Google were more likely to attract the promising talent in the analytics group at the Oakland Athletics than were other MLB clubs. Efforts by sporting leagues to invest in league wide initiatives in data collection, analysis and in talent development can play an important role in reducing the upfront and ongoing cost of building state of the art analytics groups.

*Hindsight Evaluation of Decisions* is a challenge. The appropriate benchmark for evaluating whether analytics can be an important addition to player-side decision making is whether the resulting decisions made have a higher likelihood of a desired outcome than does a decision made without analytics. Like all decisions made under uncertainty, a good decision using this criteria does not guarantee a desired outcome. In a similar vein, a good outcome can occur from a flawed decision process. Outcome-based evaluation has a long tradition by sports commentators and on talk-back radio programs. Hindsight evaluations of General Manager draft decisions often focus on players they pass on and subsequently become stars. For example, Tom Brady—one of the most successful players in NFL history—was chosen by the New England Patriots in the sixth round, as the 199th pick of the 2000 NFL draft. This draft position means he was passed over by all 32 clubs multiple times. As of 2021, at age 43, he had been the quarterback on seven Super Bowl winning teams. Hindsight talent draft overlooks should be a crucible for looking into what information cues may be useful to give more weight in future decision making but not for an automatic judgement that it implies a poor prior draft decision. Importantly, luck plays an important role in achieving good outcomes as well as making good decisions.

Being exposed to trenchant hindsight-based criticism and mocking of coach or general managers and their decisions is an inherent part of their working in the sporting industry. Concern over media criticism can lead to some highly risk-averse coaches taking decisions that line up with “conventional wisdom” and not basing their decision with guidance from analytics because of fear it may have a bad outcome. Figure 4 highlights that even in 2015 a sizable number of teams were classified in the ESPN Analytics rankings as either Skeptical (27/122 = 22%) or Non-Believers (10/122 = 8%) despite much evidence that it was an important part of player-side decision making.

A third inhibitor is the *Frequent Turnover in Coaches and General Managers* at clubs. Since coaches and general managers can vary in their preferences for different types of analytics tools, this impacts the adoption of analytics. For example, the mean (median) tenures of coaches in the NFL is 4.60 (3.80) seasons while in the NBA is 3.05 (2.18) seasons, and for general managers the mean (median) tenures in the NFL are 5.27 (5.17) seasons and in the NBA are 4.82 (4.92) seasons (Foster et al., 2020). Turnover in key personnel can lead to recently adopted programs being abandoned without adequate runway to evaluate its effectiveness. The development of strong analytics programs is aided by reasonable stability in the key stakeholders of those programs.

A fourth inhibitor, *Negative Characterizations of Analytic Approaches to Decision Making*, is the reality that new innovations can appear to be intimidating to key actors who were schooled in a different era. One set of reactions is open hostility to the new innovation, which occurs in many sectors where new modes of thinking can be challenging to those whose core expertise is being challenged. For example, Charles Barkley is one frequently quoted ex-player and media commentator in this camp. In 2015, in a critique of Daryl Morley (general manager) and the Houston Rockets he commented: “Analytics don't work at all. It's just some crap that people who were really smart made up to try to get into the game because they had no talent…Analytics don't work” (Golliver, 2015).

A fifth inhibitor is *Failure To Appreciate Shifts in Business Success Factors*. Coaches and general managers who had success with the less analytic based approaches in a prior era can rationalize that their heritage ideas continue to apply even when advances have changed the “rules of the game” for team success. The NBA shift to three point shots being a core part of playing strategy left some teams that were built around players with minimal three point scoring ability struggling to compete with teams that quickly recognized this shift (e.g., the Golden State Warriors).

A sixth inhibitor, *Privacy Issues*, applies especially to data analytics in the health and fitness areas. Unresolved issues exist as to the ownerships rights to the data collected by individual clubs about their players in areas such as training and in sleep monitoring. Tracking of career-long health and fitness data collected by clubs on athletes when they play in multiple clubs over their careers requires legal approval for sharing of data by clubs in areas which they have ownership. Many unresolved issues about data ownership and sharing protocols remain to be resolved and likely will continue to constrain research on career-based health and fitness drivers.

## MLB Case Study Highlighting the Sustained Success of the Oakland Athletics

When *Moneyball* was published in 2003, it highlighted the on-the-field success of the Oakland Athletics in the 2002 regular season when the Athletics had a 103 win/59 loss regular season record with one of the lowest payrolls in MLB. Beane became General Manager of the Athletics following the 1997 season end. In this section we probe the relative sustaining power of the Athletics strategy vis-à-vis other MLB clubs by analyzing the relationship between the rank of the 30 MLB clubs on player payroll and the rank of clubs on regular season wins over the 1998 to 2019 period. The 1998–2010 analysis involved the following steps.

**Figure 5:**
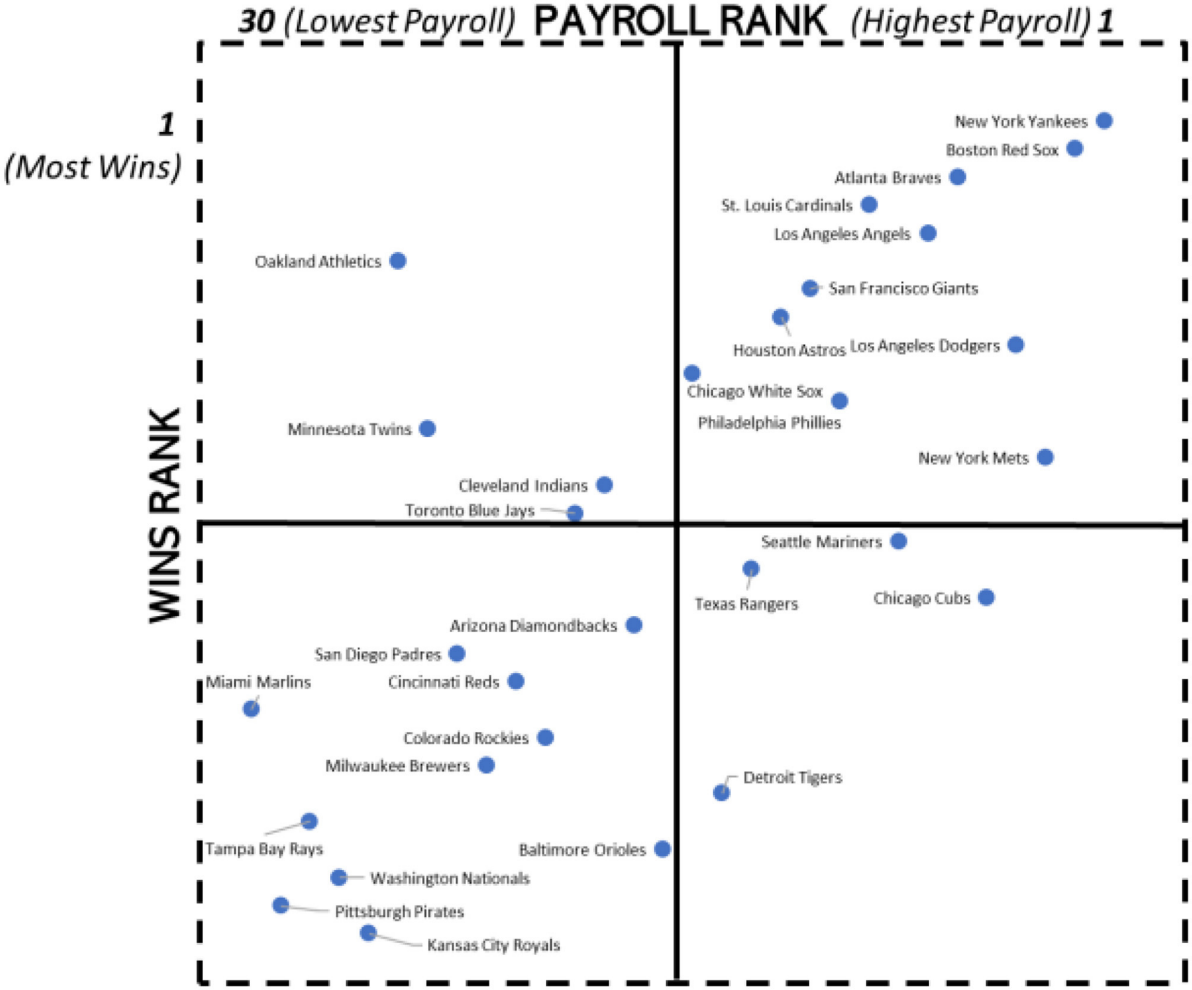
Quadrant analysis of MLB payroll rank and regular season win percentage ranks: 1998–2010.

- Step 1: For each year, we ranked the 30 MLB clubs from lowest payroll (1 = lowest payroll) to the highest payroll (30 = highest payroll)
- Step 2: For each year, we ranked the 30 MLB clubs from highest number of regular seasons wins (1 = highest number of wins) to the lowest number of wins (30 = lowest number of wins)
- Step 3: We computed the average rank on regular season wins over the 1998 to 2010 period and the average rank on MLB payrolls over the 1998 to 2010 period.
- Step 4: We plotted the 30 MLB clubs in the matrix format in Figure 5. There are four quadrants in Figure 5 are:

  ° Top Left (Quadrant 1): Clubs with below average rank on club payroll and above average rank on club wins.
  ° Top Right (Quadrant 2): Clubs with above average rank on club payroll and above average rank on club wins.
  ° Bottom Left (Quadrant 3): Clubs with below average rank on club payroll and below average rank on club wins.
  ° Bottom Right (Quadrant 4): Clubs with above average rank on club payroll and below average rank on club wins.

**Figure 6:**
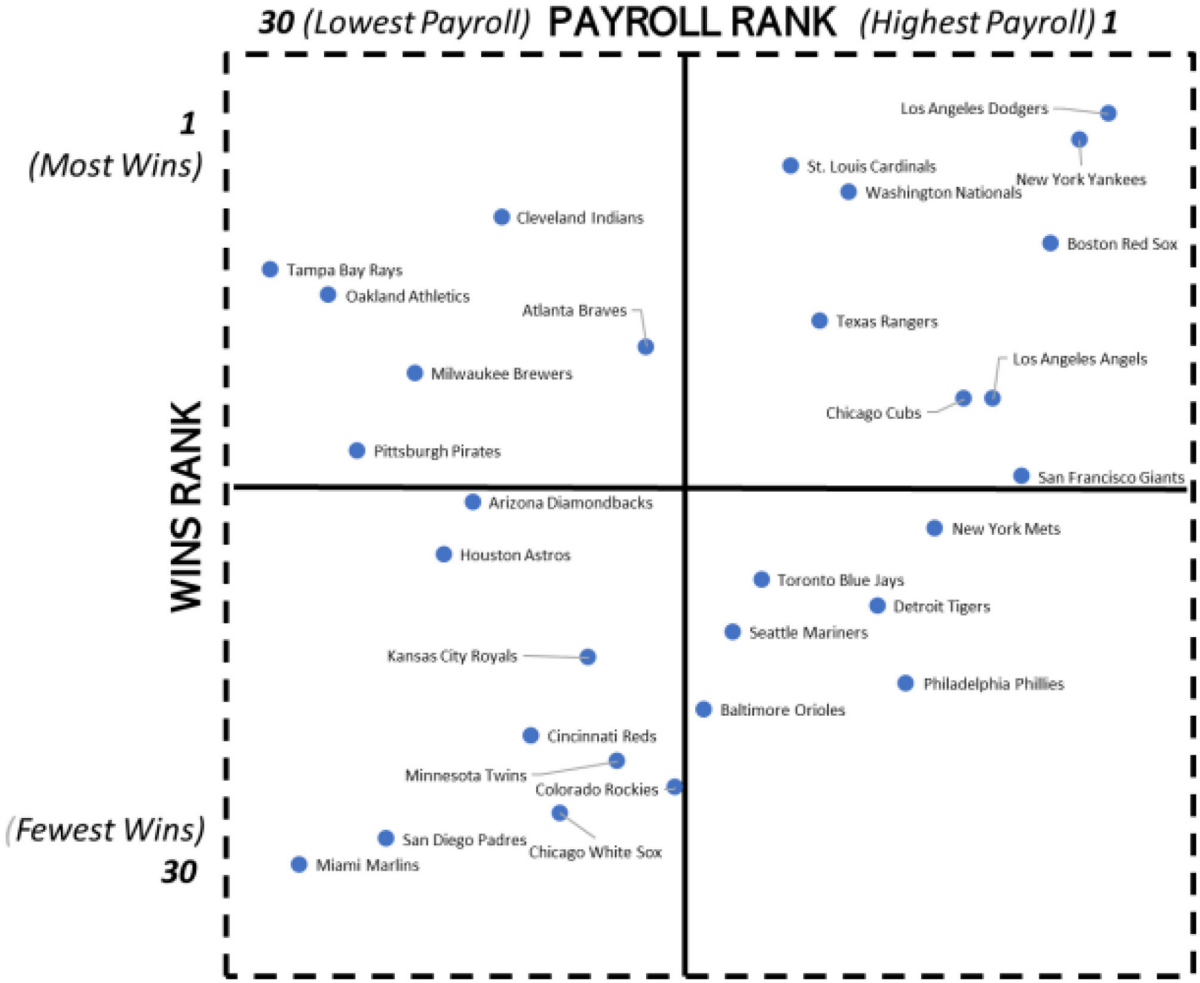
Quadrant analysis of MLB payroll rank and regular season win percentage ranks: 2011–2019.

These four steps were repeated for 2011–2019 and are reported in Figure 6.

Figures 5, 6 show the placement of the 30 MLB clubs in the 1998–2010 and 2011–2019 periods, respectively.

The economics-based hypothesis of a positive correlation between the level of club payrolls and the number of regular season wins has strong support in Figures 5, 6. The correlations based on average dollar ($) values of payrolls and the average number of regular season win in each sub-periods are reported in Table 5.

**Table 5:** Correlations of average dollar values of payrolls and average number of regular season wins in 1998–2010, 2011–2019, and 1998–2019.

	**1998–2010**	**2011–2019**	**1998–2019**
Payroll ranks/Wins ranks	0.70	0.35	0.64
Payroll $ levels/Win levels	0.72	0.54	0.72

*Data Source: NBA Reference (accessed February 10, 2021)*.

The evidence in Figures 5, 6 showcases the staying power of the Oakland Athletics over an extended period with their continued embracing of analytics. The Athletics were in the top quadrant of MLB clubs as regards the combination of low payroll and high wins in the both the two sub-periods (1998–2010 and 2011–2019) in which Beane has been a key Athletics' playing-side executive. In the 1998–2010 period, the Athletics were the most successful of the four clubs that appear in Quadrant 1 with the sixth lowest rank in payroll and the sixth highest rank in wins. It was the stunning record in the early to mid-2000's of the Oakland Athletics player payroll being relatively low while having a relatively high number of regular season wins that attracted the attention of other MLB clubs as well as clubs from other sports.

The Tampa Bay Rays move from Quadrant 3 in 1998–2010 (3rd lowest payroll/26th highest wins) to Quadrant 1 (lowest payroll/7th highest wins) in 2011-2-19 is often attributed in part to its embrace of data analytics. Tampa was an expansion MLB club in 1998. In 2004, Tampa Bay was acquired by Saul Steinberg who had been a partner in the investment banking firm Goldman Sachs. Andrew Friedman, at age 28, was appointed General Manager of Tampa starting with the 2006 season replacing a General Manager who had overseen losing seasons in every one of the first 8 years that Tampa had been a MLB club. Friedman came with a then non-traditional GM background having previously been an analyst at an investment firm (Bear Stearns) and an associate at a private-equity firm (MidMark Capital). The philosophy of seeking to identify undervalued assets and then benefitting as the broader capital market recognized their higher actual values was a well-accepted strategy in the financial investment world that both Steinberg and Friedman traveled in before linking up at the Tampa Bay Rays. While Friedman was recruited away by the Los Angeles Dodgers at the end of the 2014 season, the infrastructure he built was substantial, including a very sizable number of people in the Rays player-side analytics area (see Section Evidence on Diverse Composition of Personnel in Data Analytics Groups of MLB Clubs below). In 2020, the Rays progressed to the World Series and beat three clubs with payrolls reported by Spotrac to be much higher, including the New York Yankees (payroll 3.96 times higher) and the Houston Astros (payroll 2.92 times higher). The Rays lost the World Series to the Los Angeles Dodgers (payroll 3.83 times higher) in a six games series. As noted in Section Growth Accelerators and Growth Inhibitors of Broader and Deeper Analytics Adoption in Player-Side Decision Making, movement of elite analytic talent across clubs is one of the accelerators of analytics adoption across leagues.

## Evidence on Diverse Composition of Personnel in Data Analytics Groups of MLB Clubs

The annual Media Guides of most North American professional sporting clubs provide extensive information on their management and playing side personnel as well as the players and coaches and their support staff. Clubs vary in the level of this voluntary disclosure, especially about the playing side personnel below the General Manager, Manager and Assistant Manager levels. This section reports findings from an analysis of the 2020 Media Guides of the 30 MLB clubs. The focus was on the position titles of people in the playing-side analytics areas of these 30 clubs.

**Figure 7:**
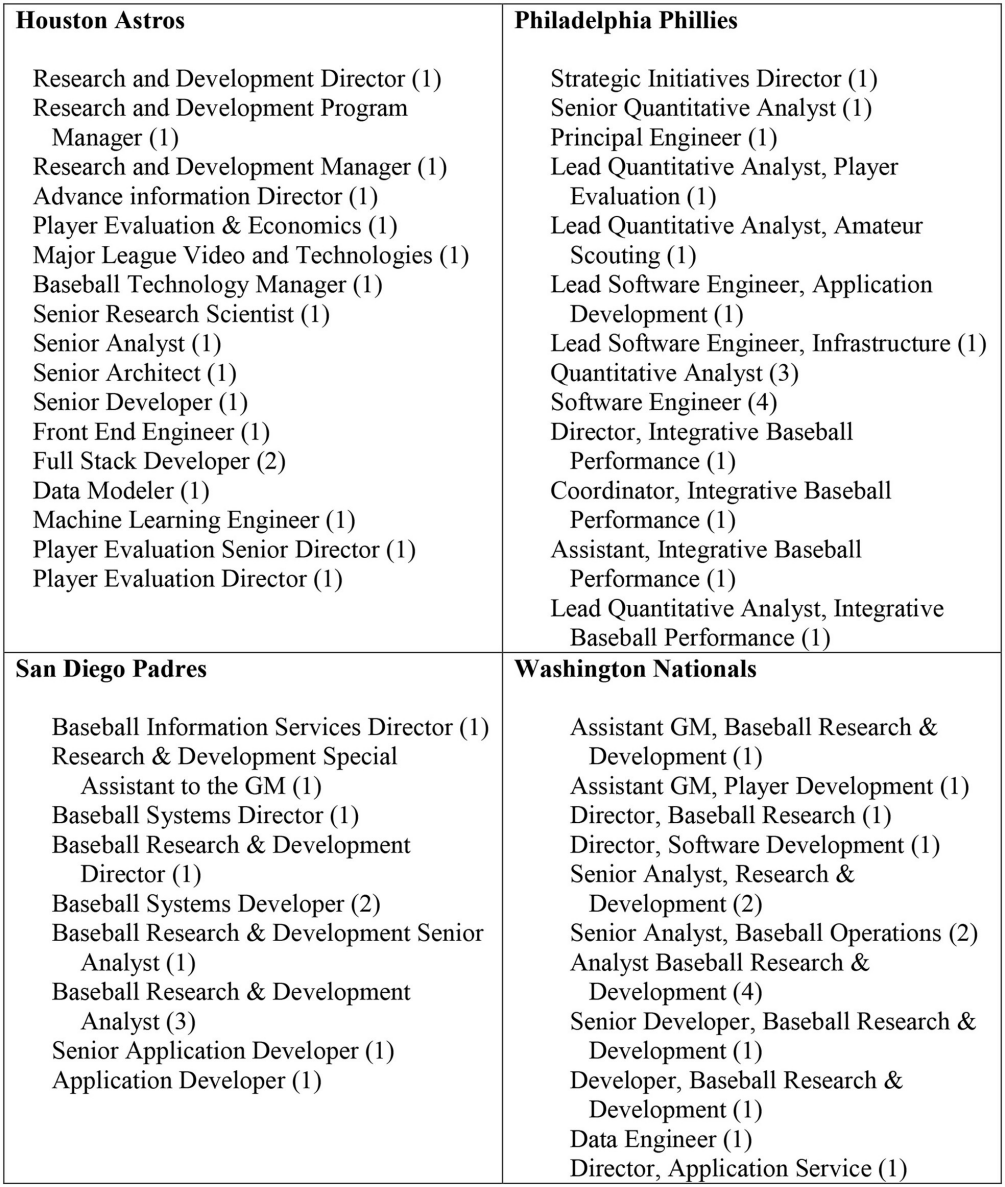
MLB club player-side analytics groups: position descriptors in 2020 media guides of four selected clubs.

One observation from the review of the Media Guides is that just seven clubs report their analytics group with names and positions as an identifiable sub-part in Baseball Operations, typically in a group called “Research and Development.” Figure 7 shares the positions reported in the identified analytics groups of four clubs —Houston Astros, Philadelphia Phillies, San Diego Padres and Washington Nationals.

The other three (of the seven) clubs are the Baltimore Orioles, Boston Red Sox, and Miami Marlins. Across the Media Guides of the 30 MLB clubs, we identified 396 positions that would fall in the ambit of data analytics activities. This identification is subjective in some cases, so the patterns observed are of a general nature rather than every tag being clearly data analytics related. The mean and median number of positions reported per club was 13.2 and 12, respectively. The three clubs with the highest number of analytics people are the Tampa Bay Rays (28), New York Yankees (22), and Texas Rangers (22). One area typically not reported in Media Guides is the many interns that are with clubs for varying lengths of time. In recent years, many clubs have expanded the number of interns in the analytics area. For instance, one club in 2020 had 6 interns on a 6 months basis, with two being retained for a longer period after that 6 months. These interns often come with undergraduate degrees with a strong emphasis of computer science, statistics and economics and had been active members of a sports analytics club at the university where they graduated.

**Figure 8:**
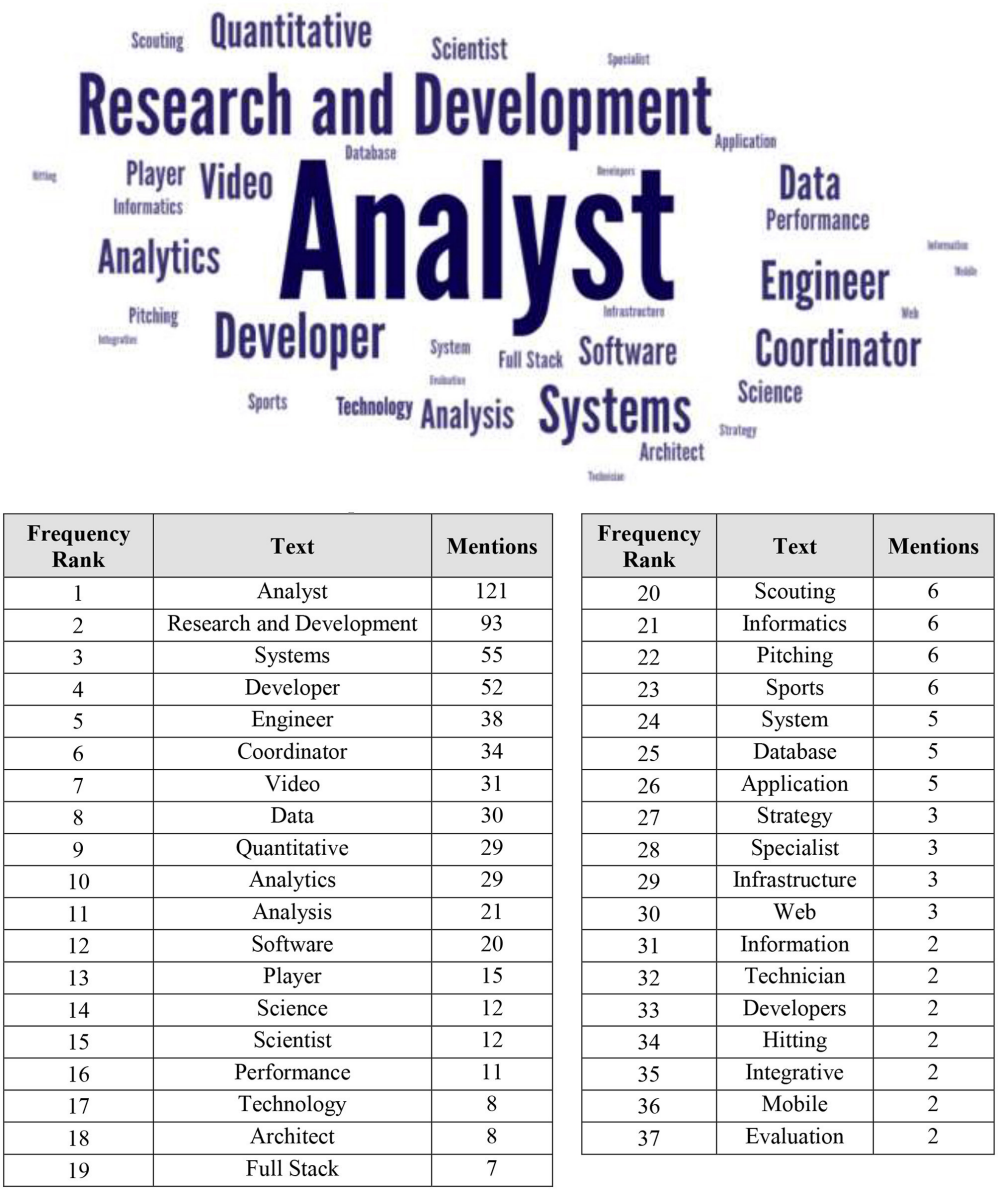
MLB club player-side analytics groups: word cloud of position descriptors from the 2020 media guides of all 30 MLB clubs.

Figure 8 presents a word cloud analysis of the position titles of the 396 people identified with player-side analytics related functions in our Media Guide review. Based on interviews with multiple clubs and on the words in Figure 8, the various functions that fall under the umbrella of player-side analytics include: (i) hardware engineers, (ii) software engineers and developers, (iii) data acquisition, (iv) data base management, (v) data scientists and analysts, and (vi) analytics report linkage with coaches, trainers, and players.

## Summary

The growing analytics movement in player-side sports is one of the most important areas of change in sporting club strategy and management. A key implication is hiring analytics talent is now at a premium. Elite levels of this talent can command high levels of compensation, in part because they are attractive to companies in many other industries. Moreover, this talent often has a non-traditional sport specific heritage or limited prior exposure to the on-field aspects of the specific sport they work in. This talent can include software engineers and data analysts that are equally attractive in many areas outside of the sports economy, including many large innovative companies as well as recently funded new technology ventures.

The likely implication of this broader adoption of analytics across clubs will be a greater emphasis on continued innovations across many areas such as player squad assembly, pre-game and within-game strategy, and health and fitness. Club and league executives will be on an ongoing treadmill to stay at an ever-more sophisticated state of the art level let alone a cutting edge level in player-side sports analytics. Key elements of these innovations will have their genesis and wide adoption in many areas beyond the sporting world.

While the focus of this paper has been on professional sports, many of the issues we have discussed have applicability to broader areas of business and society. For instance, one implication is that executive talent important in areas outside of professional sports are increasingly more relevant and valuable to many areas inside the sporting world. Hopefully this bodes well for important mutual gains occurring from a broader and deeper interaction between executives in the sporting world with executives in areas of business and society beyond sports.

## Data Availability Statement

Publicly available datasets were analyzed in this study. This data can be found here: The data used in the article came from baseballreference.com.

## Author Contributions

All authors listed have made a substantial, direct and intellectual contribution to the work, and approved it for publication.

## Conflict of Interest

The authors declare that the research was conducted in the absence of any commercial or financial relationships that could be construed as a potential conflict of interest.

## Footnotes

^1^See, e.g., the many universities ranked in U.S. News.com “Best Undergraduate Data Science Programs” for 2021. Available online at: https://www.usnews.com/best-colleges/rankings/computer-science/data-analytics-science. The top ranked programs were: 1. University of California-Berkeley, 2. MIT, 3. Carnegie Mellon, and 4. Stanford. Graduate programs at universities likewise reflect the importance that major schools now give to this area. The top ranked programs in U.S. News' “Best Business MBA Analytics Programs” for 2021. Available online at: https://www.usnews.com/best-graduate-schools/top-business-schools/business-analytics-rankings - were 1. MIT, 2. Carnegie Mellon, 3. Wharton, and 4. Georgia Institute of Technology.

^2^*Moneyball* was published in 2003 by W.W. Norton & Company, New York, NY and London. The data analytics focus of the book was further highlighted by the 2011 *Moneyball* film starring Brad Pitt and distributed by Sony Pictures.

^3^On-Base Percentage is the percent of times that a batter gets to base relative to the number of times at the plate. It includes hits by the batter, walks, and the batter being hit by a pitch that results in going to first base.

^4^Slugging Percentage is a weighted hitting metric that weights hits based on where the hitter ends up after the hit relative to the number of times at the plate. The weights are one first base, two for second base, three for third base, and four for a home run.

^5^Beane in several Stanford Graduate School of Business classes has noted that he was under the impression that Michael Lewis was writing a short article when he granted Michael Lewis “inside observation access” and wondered why it was taking Lewis so long to collect information for a short article. He subsequently concluded that the very astute Lewis had quickly surmised that there was a book in what he was observing rather just a short article. Prior to Moneyball (2003), Lewis had authored the best-selling Liar's Poker (1989).

^6^Available online at: https://www.ecaeurope.com/media/2730/eca-report-on-youth-academies.pdf.

^7^Available online at: https://www.manutd.com/en/news/detail/man-utd-celebrate-unrivalled-academy-milestone-with-4000th-game.

^8^See www.hudl.com. It is a product line of Agile Sports Technologies, Inc.

^9^Another company example in this space is Qwikcut.com.

^10^Available online at: https://www.espn.com/nfl/story/_/id/28073660/fourth-decisions-changed-good-10-years-ago-how-patriots-innovated.

^11^M. Petriello, “9 things you need to know about the shift.” MLB.com (May 16th, 2018).

^12^Available online at: https://www.cbssports.com/mlb/news/why-its-time-for-major-league-baseball-to-ban-the-shift/.

^13^An example of a meta study based on a review of 42 separate papers is Denis et al. (2018).

^14^Walsh et al. (2020).

^15^Available online at: https://nextgenstats.nfl.com/glossary.

^16^Available online at: https://www.sloansportsconference.com/about/mission-and-histor.
